# Supplementary material for: Peelable Nanocomposite Coatings: “Eco-Friendly” Tools for the Safe Removal of Radiopharmaceutical Spills or Accidental Contamination of Surfaces in General-Purpose Radioisotope Laboratories
Source: Pharmaceutics. 2022 Nov 1;14(11):2360. doi: 10.3390/pharmaceutics14112360 (PMC9695050; doi:10.3390/pharmaceutics14112360)
Supplement: Supplementary file 1 [file pharmaceutics-14-02360-s001.zip › pharmaceutics-1996161-supplementary.pdf]

pharmaceutics

Supporting information

Peelable Nanocomposite Coatings: "Eco-Friendly" Tools for the Safe Removal of Radiopharmaceutical Spills or Accidental Contamination of Surfaces in General-Purpose Radioisotope Laboratories

Traian Rotariu [1,†], Daniela Pulpea [1,†], Gabriela Toader [1,*], Edina Rusen [2,*], Aurel Diacon [1,2], Valentina Neculae [3] and John Liggat [4]

[1] Military Technical Academy "Ferdinand I", 39-49 George Cosbuc Boulevard, 050141 Bucharest, Romania
[2] Faculty of Chemical Engineering and Biotechnologies, University 'POLITEHNICA' of Bucharest, 1-7 Gh. Polizu Street, 011061 Bucharest, Romania
[3] Institute for Nuclear Research—RATEN ICN-Pitesti, 1 Street Campului, 115400 Mioveni, Romania
[4] Department of Pure and Applied Chemistry, University of Strathclyde, 295 Cathedral Street, Glasgow G1 1BX, UK
* Correspondence: nitagabriela.t@gmail.com (G.T.); edina_rusen@yahoo.com (E.R.)
† These authors contributed equally to this work.

Contents

Figure S1. Common types of surfaces in radioisotope laboratories, employed for decontamination assay: concrete **(C)**, painted metal **(PM)**,ceramic tiles **(CT)**, linoleum **(L)**, epoxy resin **(P)** 2

Figure S2. Illustration of specimens contaminated with ^{90}Sr-^{90}Y and ^{241}Am. 2

Figure S3. **(a)** Exemplification of contaminated specimens covered with one of the decontamination formulations; **(b, c)** Peeling the nanocomposite film from the decontaminated surface...................... 2

Figure S4. Recording the activity of the tested specimens.. 3

Figure S5. DSC curves obtained for the nanocomposite films... 3

Table S1. Decontamination efficacies obtained for Americium-241.. 4

Table S2. Decontamination efficacies obtained for Strontium-90 5

Figure S6. Hypothetical illustrations of the interactions established between the contaminants and the complexing agents from the decontaminating solutions... 6

pharmaceutics

MDPI

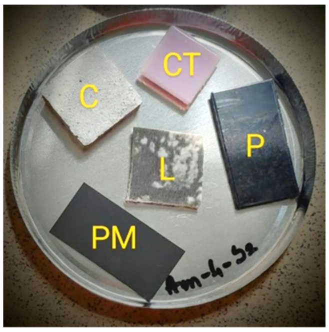

Figure S1. Common types of surfaces in radioisotope laboratories, employed for decontamination assay: concrete **(C)**, painted metal **(PM)**,ceramic tiles **(CT)**, linoleum **(L)**, epoxy resin **(P)**.

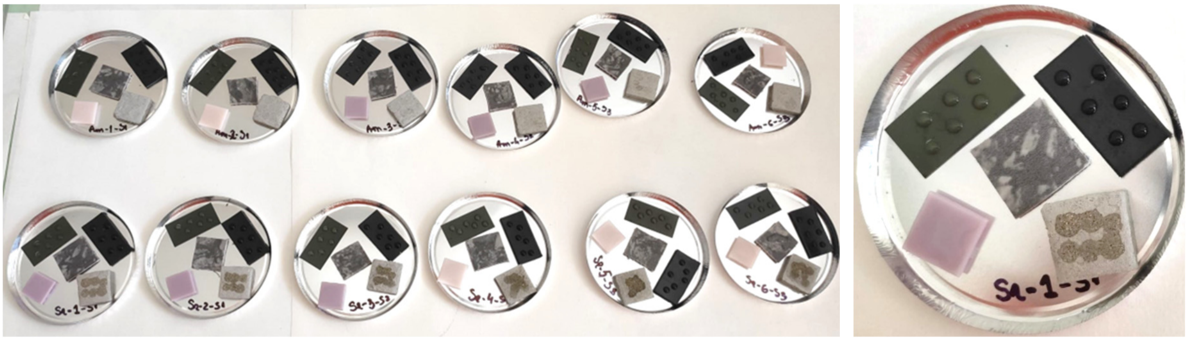

Figure S2. Illustration of specimens contaminated with ^{90}Sr-^{90}Y and ^{241}Am.

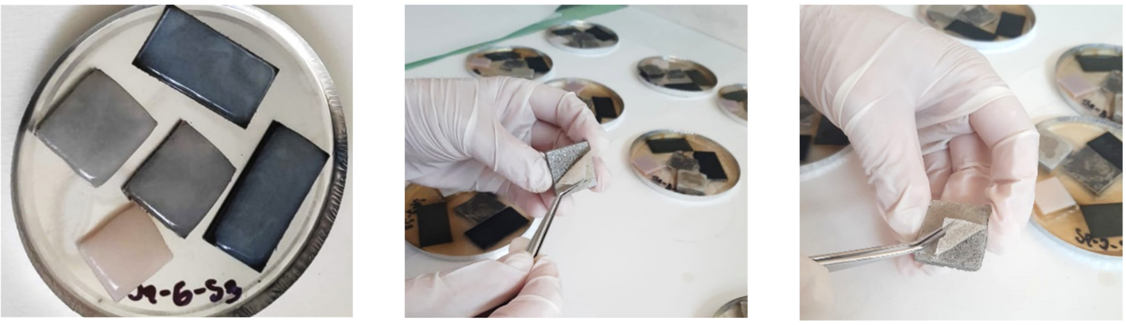

(a) (b) (c)

Figure S3. **(a)** Exemplification of contaminated specimens covered with one of the decontamination formulations; **(b, c)** Peeling the nanocomposite film from the decontaminated surface.

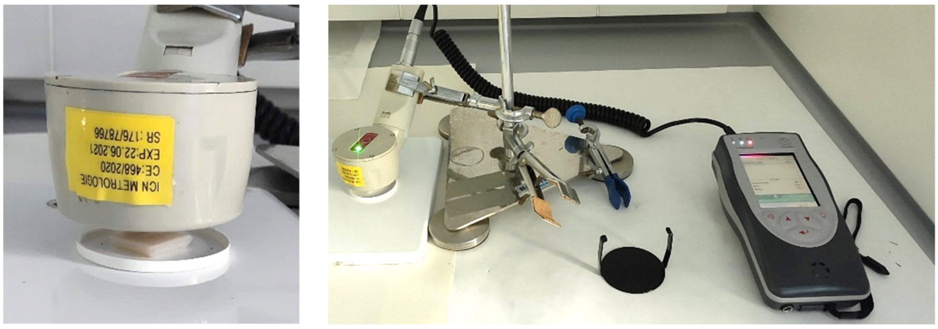

Figure S4. Recording the activity of the tested specimens.

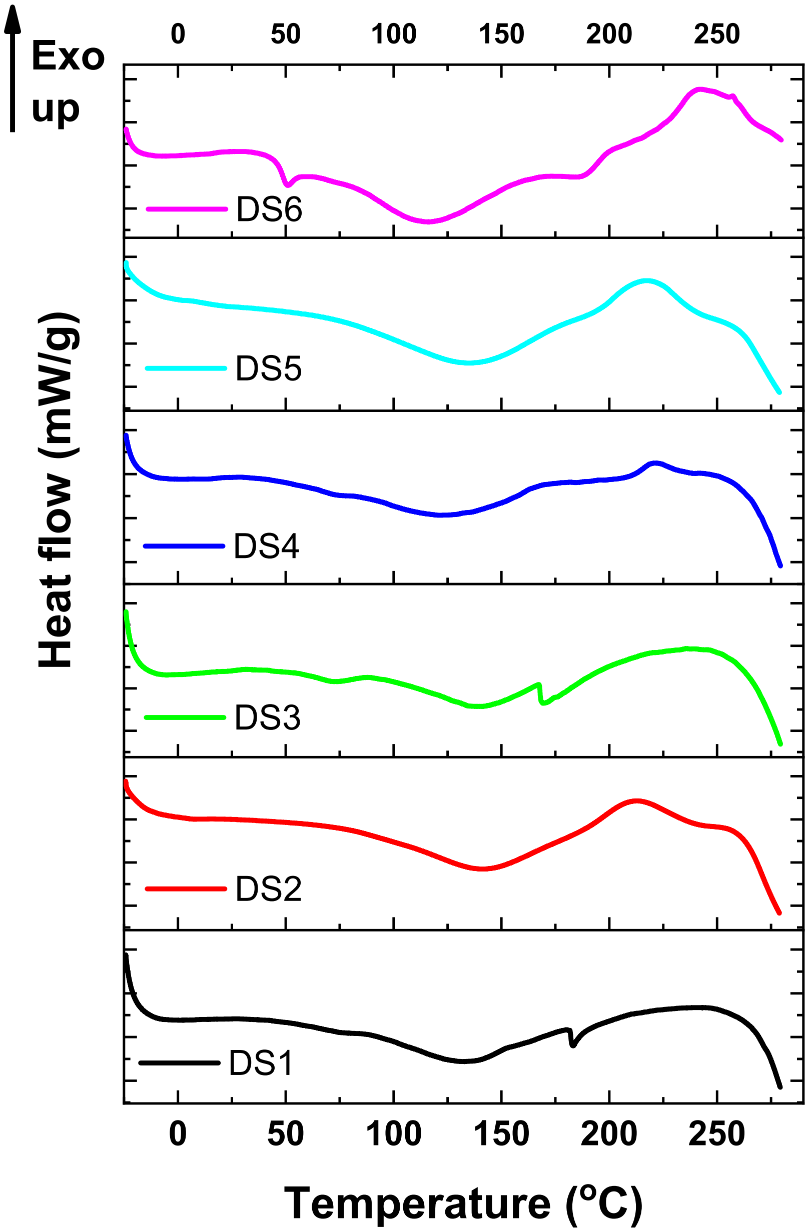

Figure S5. DSC curves obtained for the nanocomposite films.

pharmaceutics

MDPI

Table S1. Decontamination efficacies obtained for Americium-241.

Decontamination solution							DS-1								
**Surfaces**	PM			L			CT			C			P		
	Activity		DE	Activity		DE	Activity		DE	Activity		DE	Activity		DE
	Before	After		Before	After		Before	After		Before	After		Before	After	
	1810	219	87.90	1380	201	85.43	1830	208	88.63	1075	316	70.60	1550	192	87.61
	1490	232	84.43	1850	183	90.11	1660	213	87.17	1210	284	76.53	1650	192	88.36
	1643	201	87.77	1809	182	89.94	1570	203	87.07	1003	286	71.49	1862	207	88.88
**Decontamination efficacy**			**86.70**			**88.49**			**87.62**			**72.87**			**88.29**
**Standard deviation**			**1.61**			**2.16**			**0.72**			**2.61**			**0.52**
**Decontamination solutions**							**DS-2**								
**Surfaces**	PM			L			CT			C			P		
	Activity		DE	Activity		DE	Activity		DE	Activity		DE	Activity		DE
	Before	After		Before	After		Before	After		Before	After		Before	After	
	1450	224	84.55	2150	200	90.70	1730	181	89.54	1490	213	85.70	1950	197	89.90
	1850	180	90.27	1810	180	90.06	1370	202	85.26	1430	237	83.43	1590	221	86.10
	1970	211	89.29	1506	180	88.05	1690	178	89.47	1368	285	79.17	1590	160	89.94
**Decontamination efficacy**			**88.04**			**89.60**			**88.09**			**82.77**			**88.65**
**Standard deviation**			**2.50**			**1.13**			**2.00**			**2.71**			**1.80**
**Decontamination solutions**							**DS-3**								
**Surfaces**	PM			L			CT			C			P		
	Activity		DE	Activity		DE	Activity		DE	Activity		DE	Activity		DE
	Before	After		Before	After		Before	After		Before	After		Before	After	
	1970	211	89.29	1506	180	88.05	1690	178	89.47	1468	225	84.67	1590	160	89.94
	1920	206	89.27	1860	182	90.22	2090	202	90.33	1990	228	88.54	1740	159	90.86
	2270	203	91.06	2107	203	90.37	2211	208	90.59	1920	209	89.11	2380	194	91.85
**Decontamination efficacy**			**89.87**			**89.54**			**90.13**			**87.44**			**90.88**
**Standard deviation**			**0.84**			**1.06**			**0.48**			**1.97**			**0.78**
**Decontamination solutions**							**DS-5**								
**Surfaces**	PM			L			CT			C			P		
	Activity		DE	Activity		DE	Activity		DE	Activity		DE	Activity		DE
	Before	After		Before	After		Before	After		Before	After		Before	After	
	2130	250	88.26	2050	280	86.34	2100	276	86.86	1480	425	71.28	1670	203	87.84
	1980	230	88.38	1770	204	88.47	1866	225	87.94	1800	670	62.78	2124	400	81.17
	1770	180	89.83	1850	255	86.22	2080	287	86.20	1652	540	67.31	1890	347	81.64
**Decontamination efficacy**			**88.83**			**87.01**			**87.00**			**67.12**			**83.55**
**Standard deviation**			**0.71**			**1.04**			**0.72**			**3.48**			**3.04**

Activity – [counts/second]; Decontamination efficacy – [%]

Table S2. Decontamination efficacies obtained for Strontium-90.

Decontamination solution	DS-1														
**Surfaces**	PM			L			CT			C			P		
	Activity		DE	Activity		DE	Activity		DE	Activity		DE	Activity		DE
	Before	After		Before	After		Before	After		Before	After		Before	After	
	5480	245	95.53	2290	480	79.04	5430	219	95.97	5420	2730	49.63	5222	403	92.28
	5530	366	93.38	3390	597	82.39	5730	228	96.02	4770	2830	40.67	4740	256	94.60
	5689	379	93.34	3750	734	80.43	5682	302	94.68	4900	2670	45.51	5170	460	91.10
**Decontamination efficacy**			**94.08**			**80.62**			**95.56**			**45.27**			**92.66**
**Standard deviation**			**1.02**			**1.37**			**0.62**			**3.66**			**1.45**
**Decontamination solutions**	**DS-2**														
**Surfaces**	PM			L			CT			C			P		
	Activity		DE	Activity		DE	Activity		DE	Activity		DE	Activity		DE
	Before	After		Before	After		Before	After		Before	After		Before	After	
	6130	327	94.67	6120	360	94.12	6610	221	96.66	6370	3220	49.45	5320	222	95.83
	6670	241	96.39	5070	383	92.45	6000	206	96.57	4550	2730	40.00	5800	203	96.50
	6880	257	96.26	6590	430	93.47	6250	255	95.92	5860	3410	41.81	5540	460	91.70
**Decontamination efficacy**			**95.77**			**93.35**			**96.38**			**43.75**			**94.67**
**Standard deviation**			**0.78**			**0.69**			**0.33**			**4.10**			**2.12**
**Decontamination solutions**	**DS-3**														
**Surfaces**	PM			L			CT			C			P		
	Activity		DE	Activity		DE	Activity		DE	Activity		DE	Activity		DE
	Before	After		Before	After		Before	After		Before	After		Before	After	
	6510	799	87.73	6150	476	92.26	6840	245	96.42	4950	2040	58.79	5680	317	94.42
	6410	445	93.06	5980	480	91.97	7220	219	96.97	4790	2260	52.82	5810	403	93.06
	6700	680	89.85	5860	502	91.43	6580	258	96.08	4560	1970	56.80	5760	520	90.97
**Decontamination efficacy**			**90.21**			**91.89**			**96.49**			**56.13**			**92.82**
**Standard deviation**			**2.19**			**0.34**			**0.37**			**2.48**			**1.42**
**Decontamination solutions**	**DS-5**														
**Surfaces**	PM			L			CT			C			P		
	Activity		DE	Activity		DE	Activity		DE	Activity		DE	Activity		DE
	Before	After		Before	After		Before	After		Before	After		Before	After	
	6880	250	96.37	5940	220	96.30	6260	202	96.77	4800	2060	57.08	6220	305	95.10
	6930	282	95.93	5600	320	94.29	5745	202	96.48	4780	2550	46.65	6630	240	96.38
	5990	208	96.53	6350	280	95.59	5790	245	95.77	4390	2360	46.24	5620	330	94.13
**Decontamination efficacy**			**96.27**			**95.39**			**96.34**			**49.99**			**95.20**
**Standard deviation**			**0.25**			**0.83**			**0.42**			**5.02**			**0.92**

Activity – [counts/second]; Decontamination efficacy – [%]

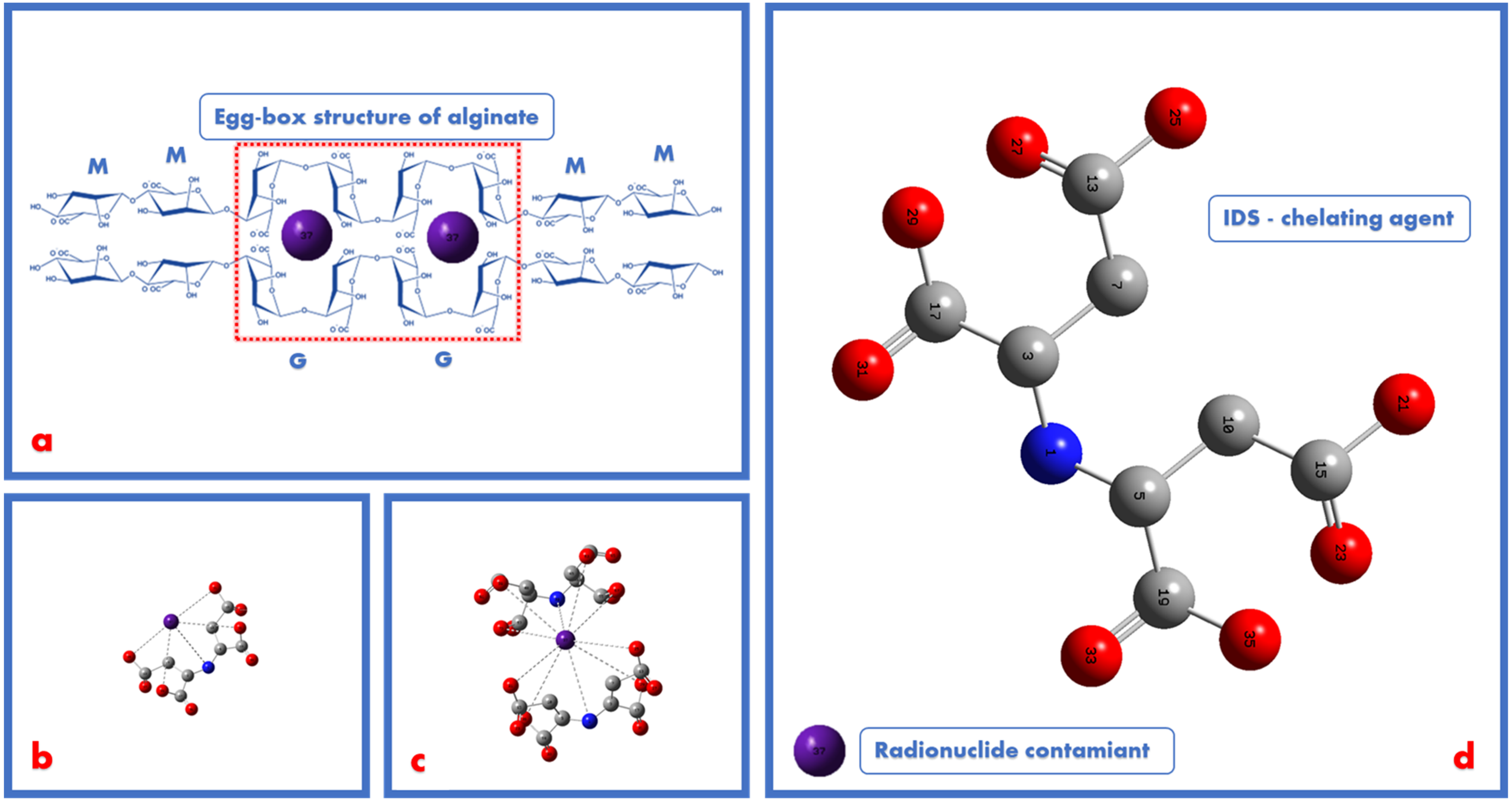

Figure S6. (**a–d**) Hypothetical illustrations of the interactions established between the contaminants and the complexing agents from the decontaminating solutions.
